# A High Throughput Approach to Reconstruct Partial-Body and Neutron Radiation Exposures on an Individual Basis

**DOI:** 10.1038/s41598-020-59695-9

**Published:** 2020-02-19

**Authors:** Igor Shuryak, Helen C. Turner, Jay R. Perrier, Lydia Cunha, Monica Pujol Canadell, Mohammad H. Durrani, Andrew Harken, Antonella Bertucci, Maria Taveras, Guy Garty, David J. Brenner

**Affiliations:** Center for Radiological Research, Columbia University Irving Medical Center, New York, NY USA

**Keywords:** Biophysics, Mathematics and computing

## Abstract

Biodosimetry-based individualized reconstruction of complex irradiation scenarios (partial-body shielding and/or neutron + photon mixtures) can improve treatment decisions after mass-casualty radiation-related incidents. We used a high-throughput micronucleus assay with automated scanning and imaging software on *ex-vivo* irradiated human lymphocytes to: a) reconstruct partial-body and/or neutron exposure, and b) estimate separately the photon and neutron doses in a mixed exposure. The mechanistic background is that, compared with total-body photon irradiations, neutrons produce more heavily-damaged lymphocytes with multiple micronuclei/binucleated cell, whereas partial-body exposures produce fewer such lymphocytes. To utilize these differences for biodosimetry, we developed metrics that describe micronuclei *distributions* in binucleated cells and serve as predictors in machine learning or parametric analyses of the following scenarios: (A) Homogeneous gamma-irradiation, mimicking total-body exposures, *vs*. mixtures of irradiated blood with unirradiated blood, mimicking partial-body exposures. (B) X rays *vs*. various neutron + photon mixtures. The results showed high accuracies of scenario and dose reconstructions. Specifically, receiver operating characteristic curve areas (AUC) for sample classification by exposure type reached 0.931 and 0.916 in scenarios A and B, respectively. R^2^ for actual *vs*. reconstructed doses in these scenarios reached 0.87 and 0.77, respectively. These encouraging findings demonstrate a proof-of-principle for the proposed approach of high-throughput reconstruction of clinically-relevant complex radiation exposure scenarios.

## Introduction

The need for *high-throughput* automated biodosimetry in response to a large-scale radiological event such as improvised nuclear device (IND) detonations stems from several considerations^1–10^. The first task is triage in a non-hospital setting, which is crucial for preventing treatment locations from being overwhelmed^11,12^. Second, it is critical to quantitatively reconstruct the radiation dose that the individual received to identify among an exposed population those individuals who are most likely to develop acute or late radiation injury and therefore require medical treatment. The third task is to convey credible information about radiation doses to potentially exposed individuals as quickly as possible^13^. Importantly, situations where radiation biodosimetry can prove useful are not limited to homogeneous total-body photon exposures, but include more complex exposure scenarios. Such scenarios are: (A) partial-body exposure, due to shielding of some body parts by dense structural materials or vehicles^14^. (B) Mixtures of densely ionizing neutrons and sparsely ionizing gamma rays, with the radiation quality and type of exposure varying between individuals^15^. Whereas low-throughput approaches were explored previously (discussed in^16,17^) for dealing with these situations, we sought to implement a truly high-throughput system.

The dicentric chromosome (DCA) and the cytokinesis-block micronucleus (CBMN) assays represent established and robust radiation biodosimetry tools because of low background yields in unirradiated individuals and reliable dose responses after exposure to ionizing radiation. High-throughput automated approaches, such as Metafer’s MNScore and DCScore scanning and imaging software (MetaSystems, Althaussen, Germany), can address these needs by performing the CBMN or the DCA assays with high speed and accuracy^18–20^.

In the current study, we used CBMN data generated from a series of experiments to evaluate micronuclei (MN) yields in peripheral blood lymphocytes exposed *ex vivo* to photons and neutrons. Our intention was to utilize the information contained in the *shapes of probability distributions* for micronuclei/binucleated cell counts in peripheral blood lymphocytes to develop a CBMN-based methodology for *high-throughput* automatic discrimination of *complex irradiation scenarios* like partial-body shielding and/or neutron + photon mixtures from simpler exposures such as homogeneous total-body photon irradiation^2,21,22^. Such a capability for rapid individualized reconstruction of exposure type on a large scale is important for making appropriate triage and treatment decisions in mass casualty situations. CBMN data were selected here as an example for the proof of principle study, and future analyses using the same conceptual rationale on DCA data could be possible. The methodology proposed here was not related to cell morphology, but to mathematical descriptors of probability distribution shapes for micronuclei per cell.

The mechanistic background for our analysis is that, compared with total-body homogeneous photon irradiations, neutrons produce more heavily-damaged lymphocytes with multiple micronuclei/cell, whereas partial-body exposures produce fewer such lymphocytes. In other words, irradiation scenarios with the same average dose can be discriminated based on differences in the *probability distributions* of damage per cell. The shapes of these probability distributions can be readily assessed for micronuclei because counts ≥2 per cell, or even ≥4 per cell, are observed at relevant doses with sufficient frequency to achieve statistical power at practical sample sizes.

### Significance of neutrons

A likely mass-irradiation scenario is a gun-type IND detonation using enriched uranium^23^. Here, the prompt acute exposure will consist of gamma rays combined with a device-dependent dose of fast neutrons^24^. The contribution of neutrons can constitute from 24 to 51% of the total prompt radiation dose at a distance of 1 km^25^. Due to the high relative biological effectiveness of neutrons for causing cytogenetic damage^26,27^, the neutron dose is expected to contribute roughly 4 times the damage of an equivalent photon dose^26^. Consequently, these neutron components are likely to have a profound impact on radiation-induced disease type and progression^28,29^. It is also likely that different countermeasures will be required for neutron-induced vs photon-induced disease^30^

### Significance of partial body exposures

A significant proportion of individuals exposed to the initial blast from an IND will be exposed non-homogeneously, to a partial body exposure, due to shielding by objects like vehicles and building materials^23^. By contrast, radioactive fallout is likely to result in a more homogenous exposure but decreases over time, approximately following a power function called “the 7:10 rule”^31,32^. Partial body exposure has important consequences in terms of medical countermeasures and disease progression^33^. For example, the hematopoietic system can recover much better after high-dose irradiation when part of the body containing bone marrow (*e.g*. one or more limbs) is shielded^34^. In animal studies, even 5% bone marrow shielding results in a large increase in survival from hematopoietic acute radiation syndrome (H-ARS)^35^ and can also profoundly affect the gastrointestinal (GI) syndrome^36^. A simple biodosimetric dose reconstruction that estimates a single dose number assumes uniform irradiation, and would thus generate incorrect results: overestimate the risk for hematopoietic acute radiation syndrome (H-ARS) and underestimate the risk for later disease in the organs that were irradiated.

### Current approaches for evaluating complex exposures

There is a large body of literature on various biodosimetry approaches for estimating radiation doses in various exposure scenarios based on micronuclei yields and other cytogenetics markers like dicentric chromosomes or micronuclei^37–56^. Biodosimetric panels using other types of biomarkers are also being developed^57–69^. Cytogenetic damage per cell distribution shapes are commonly modeled by Poisson, Negative Binomial or Neyman distributions^37,40,70–72^, and it is well known that these shapes can change depending on exposure type due to the shielding and/or differences in radiation track structure and energy deposition patterns. For example, densely ionizing radiations like neutrons tend to produce “overdispersed” distributions of cytogenetic damage, where the ratio of variance/mean becomes significantly higher than in a standard Poisson distribution (*e.g*. modeled by Dolphin’s contaminated Poisson approach)^73–78^. Partial-body exposures also tend to produce overdispersion because even if the damage distribution for a homogeneous exposure is Poisson, the contribution from a shielded fraction of the body that received a much lower dose would cause the distribution to become a mixture of two or more Poissons with different means^79,80^. Although the methodologies for analyzing these phenomena differ (*e.g*. frequentist *vs*. Bayesian techniques), a common popular approach is to fit selected probability density functions (*e.g*. Zero-Inflated Poisson or Negative Binomial) to the data^45,81,82^. The best-fit parameters and their uncertainties are then used to estimate the outcomes of interest. These approaches can be applied to high-throughput assays^2,22^.

### Potential of machine learning techniques

Importantly, the methodologies described above rely on parametric regression, such as linear or linear quadratic functions, to describe the radiation response. Machine learning, which produced powerful advances in multiple fields, is now entering the field of radiation exposure estimation^83,84^. To our knowledge, ensemble machine learning techniques such as random forests (RF) and generalized boosted regression models (GBM)^85,86^ have not yet been used for high-throughput cytogenetics-based radiation biodosimetry applications.

Both parametric and machine learning regression approaches have specific advantages and disadvantages. Parametric models are easily interpretable because each fitted coefficient has a specific meaning for relating a given predictor or predictor combination to the outcome(s). RF and GBM can be more complicated to interpret because they consist of multiple (usually >100) decision trees. However, RF and GBM tend to be more flexible than parametric models in describing nonlinear dependences and interactions between predictors, and therefore tend to be more accurate.

Ensemble methods like RF and GBM train and test multiple models of a given type on randomly-selected subsets of the analyzed data set and combine the results, thereby generating more robust and accurate predictions than those obtainable using a single model^86^. RF uses decision trees as base models, and employs “bagging” and tree de-correlation approaches to improve performance. The bagging (bootstrapping and aggregation) procedure involves generating bootstrapped samples and using a random subsample of the features for each fitted decision tree. Decision trees have some very useful properties for analyzing data set types such as those in the current study. For example, they are not sensitive to outliers and to the presence of many weak or irrelevant predictors. They are also unaffected by monotonic (*e.g*. logarithmic) transformations of the data. RF readily allows for multivariate analysis with more than one outcome variable and a common set of predictor variables. All of these properties can potentially prove useful in biodosimetry applications. GBM also uses decision trees, but the trees are averaged by boosting rather than bagging. Boosting involves iterative fitting of trees: the data are reweighted so that the next trees focus more strongly on those data points on which previous trees performed the worst. GBM readily accommodates different types of error distributions, e.g. Gaussian for continuous data and Bernoulli for binary data.

### Study design

**Figure 1:**
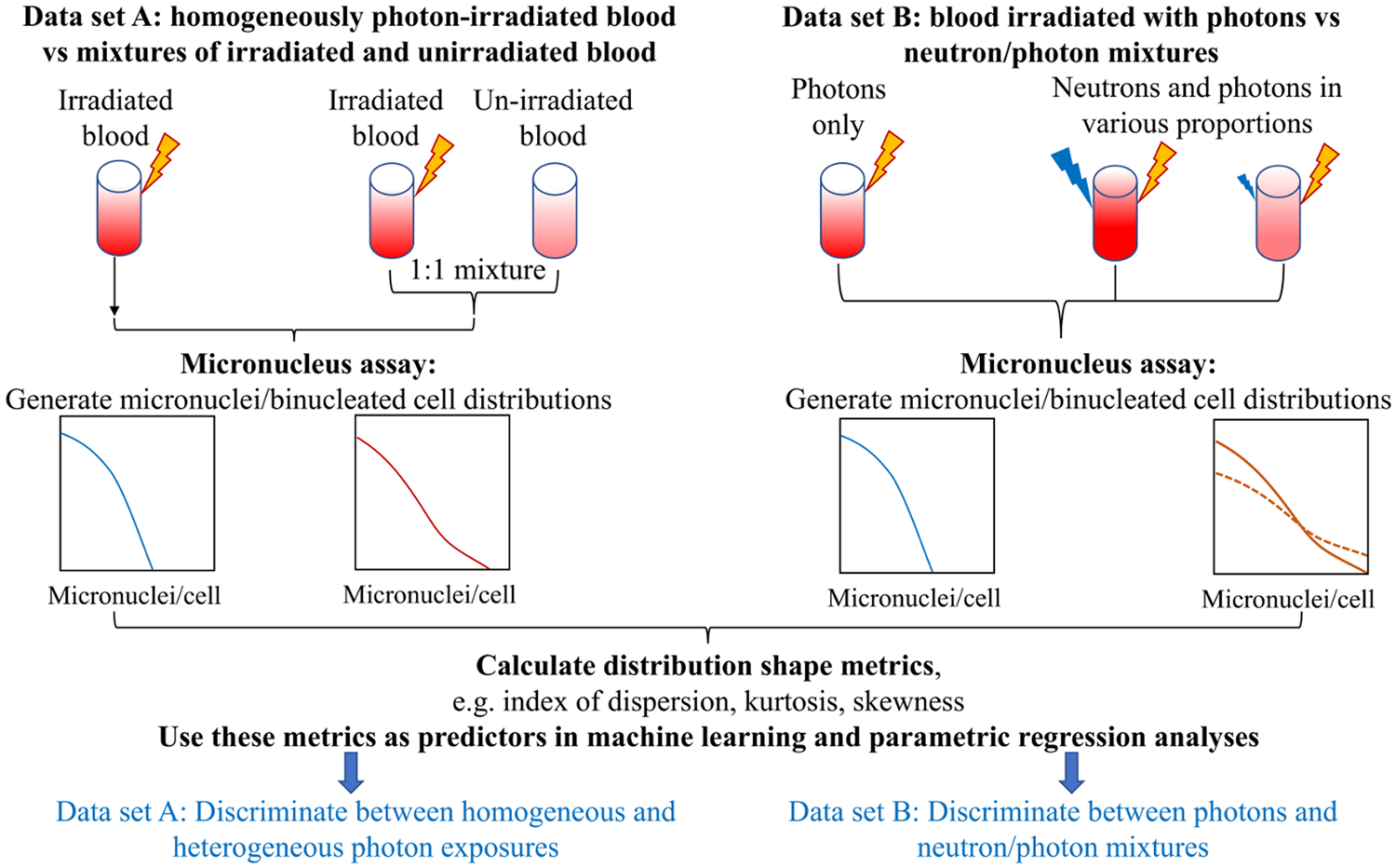
A schematic representation of our study design aimed at developing new computational methods for discriminating between triage-relevant simple and complex radiation exposure scenarios. We used *ex vivo* irradiated human blood to generate two data sets (**A**,**B**), and analyzed each of them using a novel application of machine learning techniques. The data sets and analysis methods are described in detail in the Materials and Methods section. Yellow lightning symbols indicate photon irradiation, and blue ones indicate neutron irradiation of blood samples. Curves of various colors indicate probability distributions of micronuclei per cell, where the y-axis is probability density. Solid *vs*. dashed lines indicate the effects of different neutron proportions. These schematic distributions are intended to illustrate that complex exposure scenarios, such as mixtures of irradiated and unirradiated blood, or photon + neutron exposures, produce larger “tails” (*i.e*. larger probabilities of multiple micronuclei per cell) than simple exposures.

In this work, we employed machine learning approaches (RF, GBM) in a novel role, using the *shape* of the probability distribution of micronuclei per binucleated cell as a source of information for discriminating between simple and complex radiation exposure scenarios, *e.g*. total-body *vs*. partial-body photon exposures, or *vs*. neutron + photon mixtures. Specifically, using a high-throughput CBMN assay^26,87^ we wish to evaluate on an individual basis: 1) the photon and the neutron doses and the fraction of neutrons in the total dose after a mixed exposure, 2) whether there was indeed a partial body exposure. Our study design (shown schematically in Fig. 1) consisted of using fresh human peripheral blood samples irradiated *ex vivo* to analyze the following simple and complex exposure scenarios:

*Scenario A*. Homogeneous 0, 2, 4, or 8 Gy gamma irradiation, mimicking total-body exposures, *vs*. 1:1 mixtures of 4 or 8 Gy irradiated blood with unirradiated blood, mimicking partial-body exposures. In this data set, 4 Gy-irradiated blood mixed with unirradiated blood was intended to produce a similar mean micronuclei yield to blood irradiated with a homogeneous dose of 2 Gy. The goal of the computational biodosimetry approach in this scenario was to correctly classify such situations as homogeneous exposures *vs*. mixtures in the dose range that could cause acute radiation syndrome and/or require treatment.

*Scenario B*. Photons (0–4 Gy of x-rays) *vs*. mixtures of neutrons + photons in various proportions (up to 3 Gy neutrons). The neutron proportions were intentionally varied over a wide range to mimic various types of realistic exposure scenarios. The goal of the computational biodosimetry approach in this scenario was to distinguish neutron + photon mixtures from pure photon exposures, and to quantify the neutron contribution.

## Materials and Methods

### Blood collection and irradiation

Fresh peripheral blood samples were collected by venipuncture into 6 ml lithium-heparinized Vacutainer^®^ tubes (BD Vacutainer™, Franklin Lakes, NJ) from healthy female and male donors with informed consent as approved by the Columbia University Medical Center Institutional Review Board (IRB protocol no: AAAE-2671), and all methods were performed in accordance with the relevant guidelines and regulations. Healthy blood donor volunteers, aged between the ages of 24 and 48 years were non-smokers and in relatively good health at the time of donation with no known exposure to x rays or CT scan within the last 12 months.

#### Neutron and x-ray irradiations

These irradiations were performed at the Columbia IND Neutron Facility (CINF)^26,88,89^. Our broad-energy neutron irradiator has been designed to expose blood or small animals to neutron fields mimicking those from an IND. This spectrum, dominated by neutron energies between 0.2 and 9 MeV that mimics the Hiroshima gun-type energy spectrum at a relevant distance (1–1.5 km) from ground zero^24,26^, is significantly different from a standard reactor fission spectrum, because the bomb spectrum changes as the neutrons are transported through air. Blood aliquots (1 ml) in 1.4 ml Matrix 2D-barcoded storage tubes (Thermo Fisher Scientific, Waltham, MA) were prepared and either sham-irradiated or exposed to neutrons and x rays. Details of the IND-spectrum neutron irradiator and dosimetry have been described previously^26,89^. Briefly, the aliquoted blood samples were placed in adjacent positions on an eighteen position Ferris wheel. The wheel rotates during irradiations and maintains the sample locations at a distance of 17.5 cm and an angle of 60° from the beam’s impingement on a thick beryllium target. Neutron irradiations were performed over several runs with 15–30 μA mixed beams of protons and deuterons on the target generating a neutron dose rate of 1.3–2.6 Gy/h with a 18% concomitant dose of gamma rays. To ensure a uniform scatter dose, equivalent tubes containing water were placed in any empty positions on the wheel. Dosimetry for CINF was performed, on the day of the experiment, as described previously^26^.

For the mixed photon + neutron exposure studies, some blood samples were exposed to x rays 5–10 minutes following neutron irradiation. This was done using a Westinghouse Coronado orthovoltage x-ray irradiator running at 250-kVp and 15 mA with a 0.5 mm Cu + 1 mm Al filter (Half Value Layer 2 mm Cu). X rays were delivered at a dose rate of 1.23 Gy/min. All tested combinations of x rays and neutrons are shown in the Supplementary_data_file1 online. There were 486 blood samples in this data set, and 40 different combinations of neutron dose and photon dose (including unirradiated controls). We pooled all samples that had the same neutron and photon dose combination, and in this manner generated 40 samples for analysis.

#### Gamma ray irradiations

Irradiations for partial body exposures were performed at the Center for Radiological Research, Columbia University Irving Medical Center, New York. Blood aliquots (6 ml) in 15-ml conical bottom tubes (Santa Cruz Biotechnology^®^ Inc., Dallas, TX) were prepared and transported to a Gammacell 40 ^137^Cesium (^137^Cs) irradiator (Atomic Energy of Canada Ltd.). The blood samples were placed in a custom-built 15 ml tube holder and exposed to 0 (control), 2.0, 4.0, or 8.0 Gy of γ rays at a dose rate of 0.73 Gy/min. The ^137^Cs irradiator is calibrated annually with TLDs and homogeneity of exposure across the sample volume was verified using EBT3 Gafchromic™ film with less than 2% variation within the sample (Ashland Advanced Materials, Bridgewater, NJ). For the heterogeneous exposures, the blood samples were mixed 1:1 (0 Gy and 4 or 8 Gy). There were 96 samples in this data set (Supplementary_data_file1 online).

### Cytokinesis-block micronucleus assay

Whole blood samples from each dose point were cultured in PB-MAX™ Karyotyping media (Life Technologies, Grand Island, NY), and incubated at 37 °C, 5% CO_2_, 98% humidity. After 44 h, the media was refreshed with PB-MAX™ media supplemented with cytochalasin B (Sigma-Aldrich LLC, St. Louis, MO) at a final concentration of 6 μg/mL to block cytokinesis. This protocol, which uses small volumes of blood in multi-well plate format, is based on our earlier published protocol^20,26^. After a total incubation period of 72 h, the cells were harvested. The cells were treated with 0.075 M KCl solution (Sigma-Aldrich, St. Louis, MO) at room temperature for 10 min. After hypotonic treatment, the cells were fixed with fixative (4:1 methanol:glacial acetic acid). The fixed cell samples were stored at 4 °C (at least overnight), dropped on slides, allowed to air dry for 10 min and then stained with Vectashield^®^ mounting media containing DAPI (Vector Laboratories, Burlingame, CA). The slides were left overnight at 4 °C prior to imaging.

### Imaging analysis and micronuclei scoring

Slides were imaged using a Zeiss fluorescence microscope (Axioplan 2; Carl Zeiss MicroImaging Inc., Thornwood, NY) with a motorized stage and Zeiss 10× air objective. Quantification of micronuclei yields was performed by automatic scanning and analysis with the Metafer MNScore software (MetaSystems, Althaussen, Germany) using the Metafer classifier described in our earlier work^20^. Images were captured using a high-resolution, monochrome megapixel charge coupled device (CCD) camera. For each sample, more than 1000 binucleated cells were scored and the micronuclei distribution per cell recorded. The values reported by the Metafer software were the micronuclei counts per binucleated cell, ranging from 0 to 5. The counts in the bin labeled 5 actually represent the sum of counts with values ≥5, as outputted by the Metafer software. These counts per cell were typically low (usually 0 to 3 in each sample) and therefore the lack of detailed bin information for bins >5 is unlikely to modify the results substantially.

### Compilation of the data sets

The experimental data analyzed by this study were compiled into two data sets, labeled A and B, which are presented in the Supplementary_data_file1 online. Data set A consisted of a single experimental design with homogeneous 0, 2, 4, or 8 Gy gamma irradiation, mimicking total-body exposures, *vs*. 1:1 mixtures of 4 or 8 Gy irradiated blood with unirradiated blood, mimicking partial-body exposures. Data set B was a large compilation of blood samples exposed to IND-spectrum neutrons and neutron + photon mixtures in various proportions (up to ~82% neutrons), including one previously published sample set^26^. The goal of combining such a large number of experiments was to increase statistical power and to clarify the main patterns of interest, such as the dependences of micronuclei per cell distributions on photon and neutron contributions in the dose.

### Development of predictor sets

The main goal of this study was to develop novel methods for classifying samples by radiation exposure type: “simple” exposures like homogeneous photon irradiation, *vs*. “complex” exposures like heterogeneous (*e.g*. partial-body) photon irradiation and/or neutron + photon mixed exposures. Therefore, in data set A we compared homogeneous and heterogeneous photon irradiation, and in data set B we compared photons only with neutron + photon mixtures.

Based on the distribution of micronuclei per cell counts in each sample, we calculated several summary variables, described in Table 1, for evaluation as potential predictors of simple *vs*. complex exposure type. Heavily damaged cells are less likely to reach the binucleated state needed for micronuclei scoring, causing the total number of scored cells per sample to decrease with radiation dose. This phenomenon was the rationale for using the variable **LnSum**. The other variables listed in Table 1 were used based on our judgement of what metrics could act as reasonable potential predictors of exposure type and/or dose, combined with information about overdispersion of cytogenetic damage from complex exposure scenarios^45,73,74^.

**Table 1:** Descriptions of outcome (dependent) and predictor (independent) variables used in our analyses.

Data set	Type of variable	Name	Definition
A: Homogeneous *vs*. heterogeneous gamma ray irradiation	**Outcomes:**	**MixIndex**	Binary variable: 0 = homogeneous exposure, 1 = heterogeneous exposure
		**MeanDose**	Average dose to the sample in Gy
B: x-rays *vs*. neutron + photon mixtures		**MixIndex**	Binary variable: 0 = <10% neutrons in total dose, 1 = ≥10% neutrons
		**NeutronIndex**	Binary variable: 0 = <0.5 Gy neutrons in total dose, 1 = ≥0.5 Gy neutrons
		**Photon_dose**	Photon dose in Gy
		**Neutron_dose**	Neutron dose in Gy
Both A and B	**Predictors:**	**LnSum**	Sum of analyzed cells per sample
		**LnMean**	Mean number of micronuclei per cell
		**LnVar**	Variance of the number of micronuclei per cell
		**LnVarMean**	Variance divided by the mean
		**LnZeroFrac**	ln[1 + f_0_], where f_0_ is the fraction of cells with 0 micronuclei
		**Ln3Frac**	ln[1 + f_3_], where f_3_ is the fraction of cells with ≥3 micronuclei
		**LnFD**	Fisher dispersion index, calculated according to the following equation^99^: $LnFD=\,\mathrm{ln}[{(\frac{1}{\sqrt{2n}}[(n-1)\frac{V}{M}-n])}^{2}]$
		**SEK**	Sample excess kurtosis, calculated using the following equation, where z_i_ are standardized data values using the standard deviation based on n rather than on n − 1: $LnSEK=\,\mathrm{ln}[\frac{1}{n}\mathop{\sum }\limits_{i=1}^{n}{z}_{i}^{4}-3]$
		**LnSkew**	Sample skewness, defined as **LnSkew** = ln[m_3_/SD^3^], where m_3_ is the sample third central moment and SD is its standard deviation
		**LL_exp_Pois_dif**	The difference in maximized log likelihoods for fitting an exponential distribution to the sample data *vs*. the Poisson distribution, calculated as follows, where *k* is the micronuclei count value in the *i*-th cell: $$\begin{array}{c}LLexp=\mathop{\sum }\limits_{i=1}^{n}-(k+1)\mathrm{ln}[1+M]+k\,\mathrm{ln}\,[M]\\ LLPois=\mathop{\sum }\limits_{i=1}^{n}k\,\mathrm{ln}[M]-M-\,\mathrm{ln}\,[k!]\\ {\bf{L}}{\bf{L}}\_{\bf{e}}{\bf{x}}{\bf{p}}\_{\bf{P}}{\bf{o}}{\bf{i}}{\bf{s}}\_{\bf{d}}{\bf{i}}{\bf{f}}=({\rm{LLexp}}-{\rm{LLPois}})/{\rm{n}}\end{array}$$

The prefix “Ln” indicates natural logarithm. M is the mean, V is the variance, and n is the number of cells in the analyzed sample. The predictor variables were selected based on our judgement, combined with information about overdispersion of cytogenetic damage from complex exposure scenarios^45,73,74^.

For data set A (homogeneous gamma irradiation of *ex vivo* human blood *vs*. 1:1 mixtures of irradiated and unirradiated blood) the outcome (independent) variables were called **MixIndex** and **MeanDose**. **MixIndex** was a binary variable, where 0 indicated homogeneous irradiation and 1 indicated a mixture of irradiated and unirradiated blood. **MeanDose** was the average gamma ray dose (in Gy), defined as the dose divided by 1 + **MixIndex**. In other words, **MeanDose** for a sample of mixed blood was ½ of the dose received by the irradiated blood.

For data set B (*ex vivo* human blood irradiation with x-rays *vs*. neutron + photon mixtures) the outcome variables were called **Neutron_dose**, **Photon_dose**, **MixIndex**, and **NeutronIndex**. **Neutron_dose** and **Photon_dose** represent the dose contributions (in Gy) for each radiation type, respectively. The photon dose includes the gamma ray component of the neutron beam (~18%) and the added x-ray dose. **MixIndex** in this data set was set to 1 if **Neutron_dose**/(**Neutron_dose** + **Photon_dose**) ≥ 0.1, and set to 0 otherwise. **NeutronIndex** was set to 1 if **Neutron_dose** ≥0.5 Gy, and set to 0 otherwise. In other words, **MixIndex** = 1 indicated ≥10% neutron contribution to the total dose, and **NeutronIndex** = 1 indicated ≥0.5 Gy neutron dose. The cutoff values of 10% neutrons for **MixIndex** and 0.5 Gy for **NeutronIndex** were selected based on practical relevance and to create approximately balanced data classes (*i.e*. approximately equal numbers of samples above and below the cutoff). These outcome variables for both data sets are listed in Table 1. All parameter names starting with Ln are natural log transformed.

### Data analysis

We imported both data sets into *R* 3.5.1 software for analysis, and randomly split each of them into training and testing sets (halves). Data set A was generated from a single experiment with a balanced design, with equal numbers of samples for homogeneous and heterogeneous radiation exposures. Consequently, we used the raw samples for analysis. In contrast, data set B was compiled from multiple experiments performed over several years, using a wide variety of photon and neutron doses. It contained 486 raw blood samples, where the total number of analyzed cells per sample varied greatly (from 33 to 3561) and the representation of different neutron + photon combinations was not equal. Consequently, we pooled (summed) all samples with the same combination of photon and neutron doses using the *aggregate* function in *R*. The raw and processed data sets are contained in the Supplementary_data_file1 online.

The training half of each data set was used for model fitting and selection, and the testing half was used to assess model performances. On the training data, we generated Spearman’s correlation coefficient matrices, including all predictors and outcome variables. To analyze all outcome variables simultaneously, using the same set of predictors, we employed the multivariate random forest (RF) machine learning approach (*MultivariateRandomForest R* package, https://cran.r-project.org/web/packages/MultivariateRandomForest/index.html) on each data set^90^. The outcome variables were **MeanDose** and **MixIndex** for data set A, and **Neutron_dose**, **Photon_dose**, **MixIndex**, and **NeutronIndex** for data set B, as defined above. In data set B we also analyzed the “photon-equivalent dose”, defined as x-ray dose + RBE × neutron dose, where RBE is the neutron relative biological effectiveness. RBE was an adjustable parameter, and the analysis was performed using RF.

To focus in more detail on the main outcome variable of interest in both data sets, **MixIndex**, and to identify the strongest predictors of this variable, we also used the generalized boosted regression (GBM) algorithm^86,91^ (*gbm R* package, https://cran.r-project.org/web/packages/ gbm/index.html) with a Bernoulli error distribution, and logistic regression (LR). The RF, GBM and LR methodologies and their implementation in our study are described in Supplementary Methods and Tables.

## Results

### Analysis of data set A: homogeneous vs. non-homogeneous irradiation

#### Shape of micronucleus distribution

**Figure 2:**
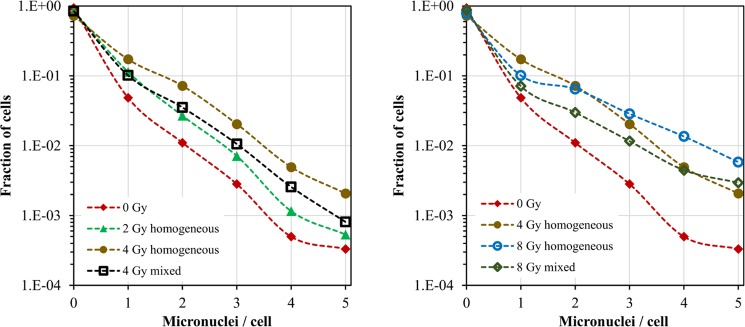
Distributions of micronuclei per binucleated cell (data set A): blood samples *ex vivo* irradiated with 0, 2, 4 or 8 Gy of gamma rays (labeled “homogeneous”), or with 1:1 mixtures of 4 Gy with 0 Gy or 8 Gy with 0 Gy (labeled “mixed”). The differences between these distributions form the basis for our analysis aimed at discriminating between homogeneous and mixed exposures. Specifically, the data for 4 Gy mixed with 0 Gy are different from those for 2 Gy homogeneous (left panel), and the data for 8 Gy mixed with 0 Gy are different from those for 4 Gy homogeneous (right panel). Each curve was based on pooled analysis of a very large number of binucleated cells (from 8,417 to 21,056).

In this data set, partial-body exposures were mimicked by mixing gamma-irradiated and unirradiated blood samples, and total-body exposures were mimicked by standard *ex-vivo* irradiation. The goal of the analysis was to use metrics related to the shape of micronuclei per binucleated cell distributions to distinguish between homogeneous and mixed exposures. Differences in micronuclei/cell distributions between these exposure scenarios were apparent upon visual inspection of the pooled data (Fig. 2). For example, the distribution of micronuclei per cell for a 1:1 mixture of 4 Gy irradiated blood with 0 Gy unirradiated blood was different from the distribution for blood irradiated with 2 Gy of pure gamma rays (Fig. 2), despite the fact that the mean micronuclei yields per binucleated cell were similar for these two scenarios (0.20 *vs*. 0.22, respectively).

**Figure 3:**
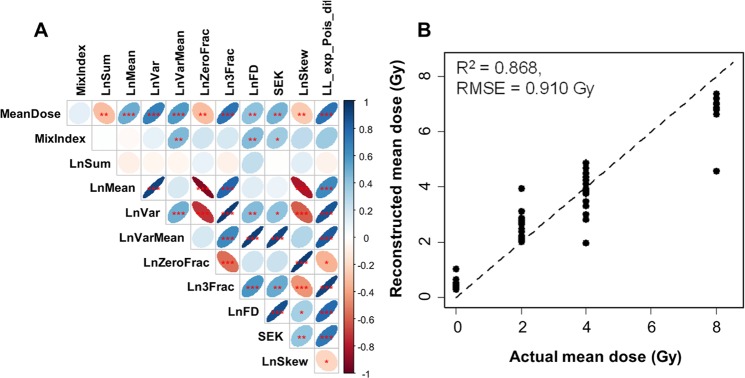
Analysis results summary for data set A: *ex vivo* human blood irradiated with homogeneous gamma ray doses *vs*. 1:1 mixtures of irradiated and unirradiated blood. (**A**) Matrix of Spearman’s correlation coefficients (pairwise, without correction for multiple testing) between predictors and outcome variables. The meanings of all variables are provided in Table 1, and a color-coded correlation scale is provided on the right of the plot. Blue ellipses represent positive correlations, and red ones represent negative correlations. Darker color tones and narrower ellipses represent larger correlation coefficient magnitudes. Red star symbols indicate statistical significance levels: ***indicates p < 0.001, **indicates p < 0.01, *indicates p < 0.05, no stars indicates p > 0.05. These p-values here are intended only for visualization: due to multiple comparisons, only 3 star significance levels are likely to indicate strong associations. Blank squares indicate correlation coefficients close to zero. (**B**) Comparison of actual mean doses with reconstructed values by RF. Circles represent data points, and the line represents theoretically perfect 1:1 correlation.

These differences were also reflected in the correlation matrix of predictors and outcomes (Fig. 3A). This matrix provides a convenient visualization of how all the analyzed variables are related to each other. As expected, the binary variable **MixIndex**, which indicated heterogeneous (mixed) *vs*. homogeneous exposure, was positively correlated with metrics of overdispersion: **LnVarMean**, **LnFD**, and **SEK** (Fig. 3A). In other words, overdispersed micronuclei/cell distributions with large “tails” were associated with heterogeneous exposures, whereas homogeneous irradiation was associated with lower variance/mean ratios and smaller “tails”.

The average dose received by each blood sample (**MeanDose**) was positively correlated with metrics for total damage, *e.g*. the mean micronuclei yield (**LnMean**) and the fraction of cells with ≥3 micronuclei (**Ln3Frac**), and negatively correlated with the sum of all analyzed cells (**LnSum**) and with the fraction of cells with zero micronuclei (**LnZeroFrac**) (Fig. 3A). In other words, the mean micronuclei yield, the total number of cells that made it to the binucleated stage, and the fraction of cells with no micronuclei were correlated with the average dose received by the blood sample.

#### Classification of partial body exposures

Multivariate machine learning analysis of data set A showed very good performance for reconstructing **MeanDose** and for reconstructing heterogeneous exposures (**MixIndex**) in a binary classification (Fig. 3B, Supplementary Table 1). Specifically, the area under the receiver operating characteristic curve (AUC) for **MixIndex**, generated by RF analysis on the testing data was 0.93 (range over 300 repeats was 0.90, 0.95), which falls into the “excellent” category for ROC curve metrics^92^ (Supplementary Table 1). Univariate analyses using GBM and LR, which focused on reducing the predictor set and identifying the strongest predictors of **MixIndex**, as described in Supplementary Methods and Tables, performed in the “fair” to “good” range^92^ (Supplementary Tables 1 and 2). The retained strongest predictors were **LL_exp_Pois_dif**, **LnVarMean**, and **LnFD** according to GB, and **LnFD** and **LL_exp_Pois_dif** × **SEK** according to LR. As mentioned above, these predictors indicate distribution shapes that are overdispersed relative to Poisson and are more similar to an exponential dependence, with a large “tail” at multiple micronuclei/cell. Their specific meanings are listed in Table 1 and in the Materials and Methods section.

### Analysis of data set B: photons vs. neutron + photon mixtures

**Figure 4:**
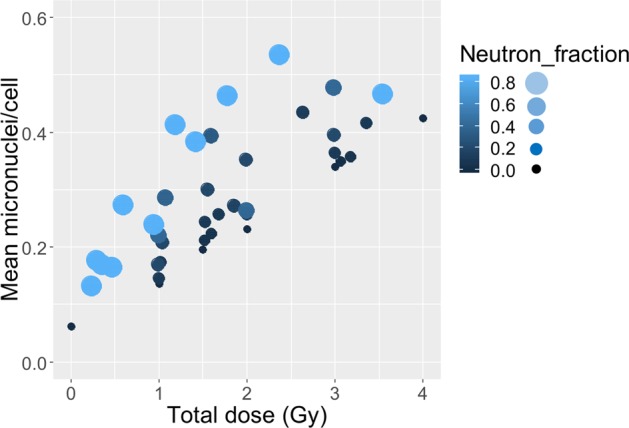
Dependence of mean micronuclei yield per binucleated cell on total radiation dose (photons + neutrons) and on the fraction of neutrons in this dose (Neutron_fraction). Larger and lighter colored circles represent a larger fraction of neutrons in the total dose.

This large data set consisted of *ex vivo* human blood samples exposed to x rays *vs*. neutron + photon mixtures in various proportions. The dependence of the mean micronucleus yield per binucleated cell on total radiation dose (photons + neutrons) and on the neutron contribution to this dose is shown graphically in Fig. 4. These data suggest that increasing the neutron contribution to the total dose notably increased the mean micronuclei yield, which is consistent with the high RBE of neutrons^26,27,93^. It was also seen that, in mixed exposures, the yield of micronuclei is given by the sum of the yield of micronuclei we would expect from the separate photon and neutron irradiations – thus the two radiation types appear to be additive with respect to micronucleus yields.

#### Shape of micronucleus per cell distribution

**Figure 5:**
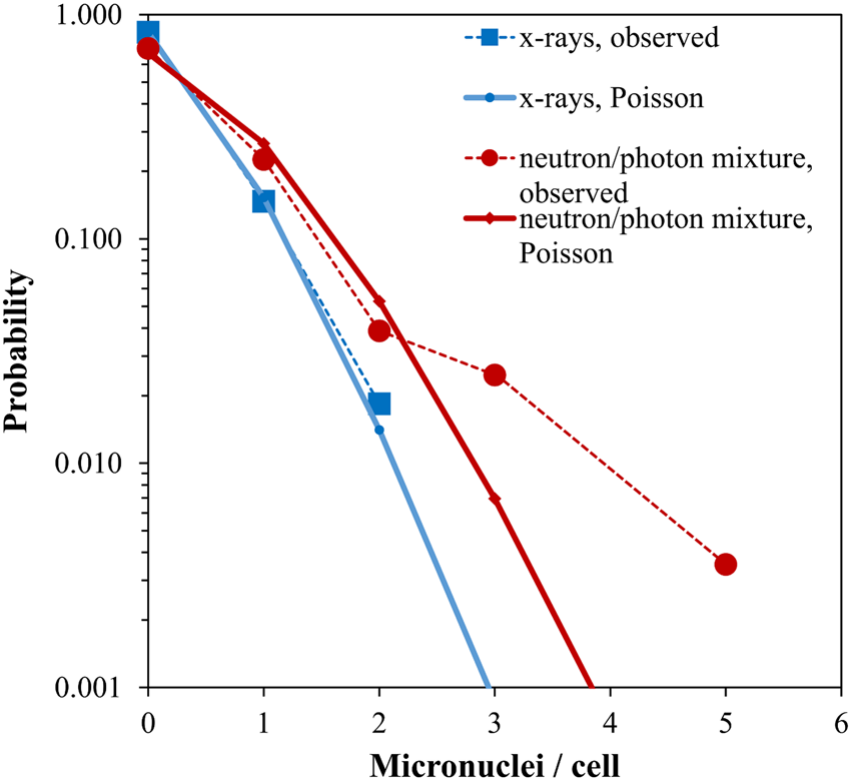
Comparison of Poisson distribution fits to micronuclei per binucleated cell data for 1.0 Gy x-rays *vs*. 1.2 Gy of a mixed neutron + photon beam that contains ~82% neutrons. The probabilities of 3–5 micronuclei per cell in the mixed beam data are much larger than those predicted by the best-fit Poisson distribution. No symbols are shown for micronuclei per cell values for which the observed counts were zero.

The presence of neutrons in the total dose also markedly alters the *shape* of the micronuclei per binucleated cell distributions. For example, Fig. 5 compares Poisson distribution fits to our micronuclei per cell data for 1.0 Gy of x-rays or 1.2 Gy of a neutron + photon beam (~82% neutrons). The x-ray data in this example are clearly much more consistent with the Poisson distribution than the neutron beam data, which have a much larger “upper tail”, *i.e*. higher than Poisson-predicted probabilities of multiple micronuclei per cell.

**Figure 6:**
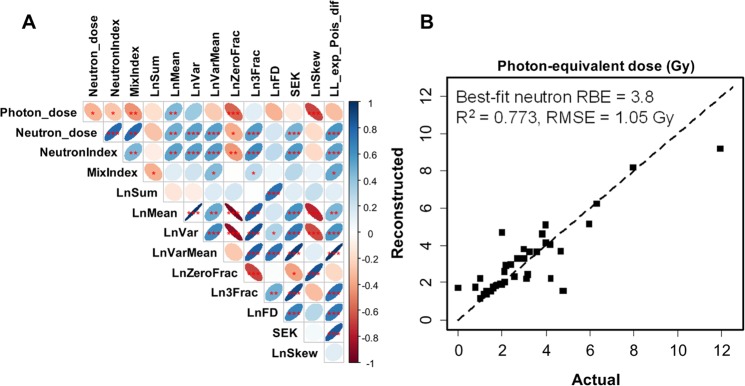
Analysis results summary for data set B: *ex vivo* human blood irradiated with x-rays *vs*. neutron + photon mixtures. (**A**) Matrix of Spearman’s correlation coefficients (pairwise, without correction for multiple testing) between predictors and outcome variables. The meanings of all variables are provided in Table 1 and in the main text. The meanings of ellipse shapes and colors are the same as in Fig. 3, and a color-coded correlation scale is provided on the right of the plot. Blank squares indicate correlation coefficients close to zero. (**B**) Comparison of actual photon-equivalent doses (defined as photon dose + RBE × neutron dose) with reconstructed values by RF. Circles represent data points, and the line represents theoretically perfect 1:1 correlation.

These effects of neutrons on the micronuclei/cell distribution are reflected in the correlation matrix of predictor and outcome variables, shown in Fig. 6A. Neutron dose was positively correlated with metrics for high damage yield (**LnMean**, **Ln3Frac**) and overdispersion (**LnVarMean**, **SEK**, **LL_exp_Pois_dif**), and negatively correlated with metrics for low damage yield (**LnSum**, **LnZeroFrac**) (Fig. 6A). Photon dose had the opposite correlation pattern regarding **LnVarMean**, **SEK** and **LL_exp_Pois_dif**, compared with neutron dose. These trends are intuitively explainable by the known overdispersion of neutron-induced damage compared with photon-induced damage^73^.

#### Classification of neutron exposures

The binary variable **NeutronIndex**, which indicated exposure to ≥0.5 Gy of neutrons, had essentially the same correlation patterns as neutron dose (Fig. 6A). The variable **MixIndex**, which indicated ≥10% of neutrons in the total dose, was most strongly positively correlated with two predictors: **LL_exp_Pois_dif** and **LnVarMean**, again suggesting that the overdispersion phenomenon is associated with neutron irradiation.

Multivariate RF analysis of data set B was quite good in reconstructing the photon-equivalent dose, defined as photon dose + RBE × neutron dose (Fig. 6B, Supplementary Table 1). The concordance between predictions and actual values was particularly close in the dose region around 2 Gy, which is important for triage decision-making (Fig. 6B). The best-fit neutron RBE value was 3.8, very similar to the previously published value of 4 for micronuclei following irradiation at CINF^26^.

**Figure 7:**
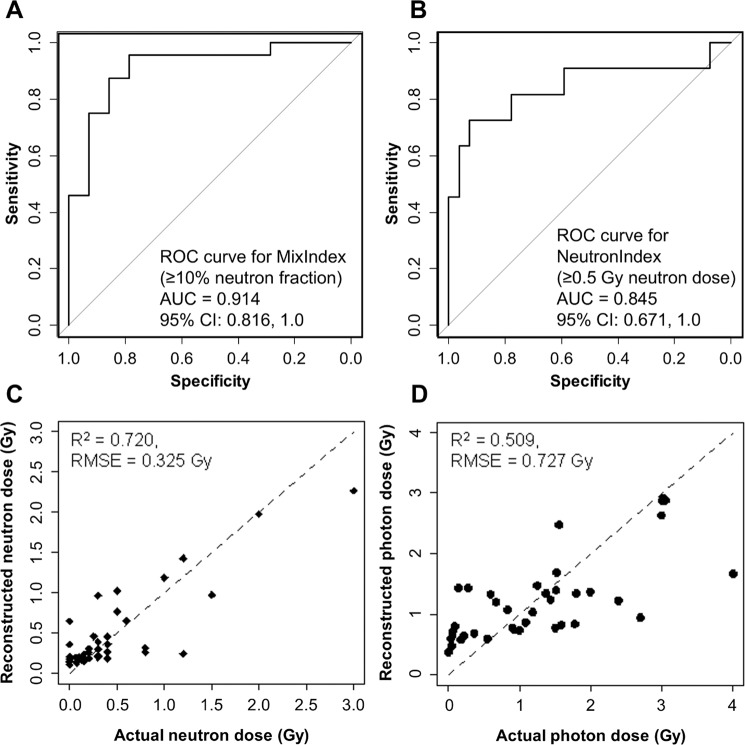
RF performance for data set B: *ex vivo* human blood irradiated with x rays *vs*. neutron + photon mixtures. (**A**) ROC curve for discriminating between exposures with ≥10% neutron fraction *vs*. those with <10% neutrons. (**B**) ROC curve for discriminating between exposures with ≥0.5 Gy neutron dose *vs*. those with <0.5 Gy neutrons. (**C,D**) Comparisons of actual and reconstructed neutron and photon doses, respectively. Circles represent data points, and the lines represents theoretically perfect 1:1 correlation.

Notably, multivariate RF was very good at detecting a neutron fraction ≥10% (**MixIndex**) and neutron doses ≥0.5 Gy (**NeutronIndex**) in binary classifications (Fig. 7A,B). The AUC values for **MixIndex** and **NeutronIndex** were 0.92 (uncertainty range 0.89 to 0.94 over 300 RF repeats) and 0.85 (0.82 to 0.88), respectively (Supplementary Table 1). These values fall into the good to excellent range for ROC curve metrics^92^. Targeted analyses using GBM and LR (described in Supplementary Methods and Tables) performed as well as RF in predicting **MixIndex**, with AUC of 0.92 (0.88, 0.96) and 0.91 (0.82, 1.0), respectively (Supplementary Tables 1 and 3). These techniques used fewer predictors: **LnVarMean**, **LL_exp_Pois_dif**, **LnSum**, **SEK**, **Ln3Frac**, and **LnZeroFrac** for GB, and **LnSum**, **LL_exp_Pois_dif** × **Ln3Frac**, and **LL_exp_Pois_dif** × **LnSum** for LR. Therefore, accurate predictions of **MixIndex** were generated using predictor groups that were indicative of overdispersion (*e.g*. **LnVarMean** and **LL_exp_Pois_dif**) and total damage yields (*e.g*. **LnSum**, **Ln3Frac**, and **LnZeroFrac**).

Quantitative reconstructions of the neutron and photon dose components (**Neutron_dose** and **Photon_dose**, respectively) were weaker, compared with the binary classifications. Neutron dose reconstructions were decent (Fig. 7C, Supplementary Table 1), and photon dose reconstructions were relatively poor (Fig. 7D, Supplementary Table 1). These results may indicate that the selected predictor set, which was focused on micronuclei/cell distribution shapes, is a sensitive qualitative indicator of complex exposure scenarios, but is less sensitive for quantifying the details of these scenarios.

## Discussion

High-throughput automatic biodosimetry is crucial for an effective response to a large-scale radiological event like an improvised nuclear device (IND) detonation^1–10^. The importance of high-throughput biodosimetry is well recognized, because traditional manual scoring assays are labor intensive, time consuming and impractical after a large-scale radiological/nuclear event^3,21,94–96^. Numerous enhanced approaches based on cytogenetic damage^37–41,43–46,48–51,54–56^ and other types of radiation biomarkers^57–59,61–69^ have been proposed and implemented. The CBMN assay is one of the simplest cytogenetic biodosimetry assays to perform and score and is also easy to automate^97^. Importantly, the shapes of micronuclei/cell probability distributions provide a rich source of information for analyses, enabling irradiation scenarios with the same average micronuclei frequencies but different damage/cell distribution patterns to be discriminated.

Such analyses are generally based on fitting parametric linear or linear quadratic dose response functions with selected error distributions (*e.g*. Poisson, Zero-Inflated Poisson or Negative Binomial)^45,79,81,82^. Here we extended the field in a different direction: we used various summary metrics like index of dispersion, skewness and kurtosis as potential predictors of complex exposure scenarios, and imported these predictors into machine learning or parametric regression methods. The conceptual basis for our approach is that micronuclei per binucleated cell distributions from complex exposures have different shapes (*e.g*. “tails”), compared with distributions from simple exposures, even when the mean micronucleus yields are the same for both scenarios. These differences in distribution shapes translated into differences in variables like index of dispersion, kurtosis and skewness (Table 1), which were used by machine learning and parametric modeling approaches to predict the outcomes of interest. To our knowledge, this approach is new and was not used previously in radiation biodosimetry, specifically for reconstructing neutron exposures.

Our results suggest that 1:1 mixtures of irradiated and unirradiated blood can be quite accurately discriminated from homogeneous irradiations (AUC >0.9 on testing data, Supplementary Table 1). Ongoing work is focusing on determination of the minimal shielded percentage that can be reliably detected. Using the same approaches, we also obtained encouraging results in discrimination of mixed exposures to photons and neutrons from pure photon exposures, *e.g*. by detecting ≥10% neutron fractions or ≥0.5 Gy of neutrons in the total dose (AUC >0.9 for the first scenario and >0.8 for the second, Fig. 7A,B, Supplementary Table 1). Of note, the dose reconstructions performed using this method reasonably estimated the measured RBE (3.8 in this work *vs*. 4 in reference^26^). Ongoing work focuses on obtaining more precise reconstructions of the neutron fractions and photon doses.

Therefore, although the two scenarios (partial body and neutron exposures) differ in experimental design and radiation doses and types, the general concept of using micronucleus distribution shape metrics as indicators of complex *vs*. simple exposure scenarios was applicable in both situations. At this stage, our results of course represent only a proof of principle because *ex vivo* blood irradiation is an “idealized” model system for partial-body and neutron + photon mixed exposures. Much more complexity is expected for realistic *in vivo* scenarios because various organs, which are (or are not) irradiated in the *ex vivo* situation can contribute to the *in vivo* responses. Furthermore, a realistic exposure may include both neutron and partial body photon exposures. These type of scenarios were not investigated in this work but will be the focus of future studies. The accuracy of applying the approaches proposed here under realistic mass-casualty conditions can probably be increased by integrating micronuclei assays with other types of radiation biomarkers (*e.g*. dicentric chromosomes, gene expression levels, blood cell counts). Several biomarkers, combined into one framework, are likely to provide more detailed and useful information than a single assay alone.

The analysis presented here used data gathered by automatic micronuclei/binucleated cell scoring. In future studies, we will enhance our high-throughput capabilities further using the second-generation Rapid Automated Biodosimetry Tool II (RABiT-II) developed by our team at the Center for Radiological Research^97,98^. Recently, we have integrated imaging flow cytometry into the RABiT-II system^2^ and showed - for the first time, to our knowledge - that high-throughput radiation biodosimetry by the CBMN assay is practical using commercial robotic systems.

## Conclusions

We demonstrate a proof of principle that a *high-throughput* automated micronucleus assay in human lymphocytes provides sufficient information to reconstruct complex exposure scenarios involving partial-body shielding or densely ionizing radiations such as neutrons, compared with homogeneous photon irradiation. The reconstruction was performed by analyzing the *shapes of probability distributions* for micronuclei per binucleated cell using a novel implementation of *machine learning* and parametric regression methods. The ability to perform such reconstructions reliably and in a high-throughput manner would be extremely useful in radiation-related mass casualty situations such as IND detonations because partial-body and/or neutron exposures can have very different clinical outcomes than homogeneous photon exposures.

## Supplementary information

Supplementary methods and tables.

Supplementary data file 1.

## References

[1] Garty G (2017). Mice and the A-Bomb: Irradiation Systems for Realistic Exposure Scenarios. Radiat. Res..

[2] Wang, Q. *et al*. Automated Triage Radiation Biodosimetry: Integrating Imaging Flow Cytometry with High-Throughput Robotics to Perform the Cytokinesis-Block Micronucleus Assay. *Radiat. Res*. RR15243.1, 10.1667/RR15243.1 (2019). 10.1667/RR15243.1 PMC6483078 30779694

[3] Jacobs, A. R. *et al*. Role of a high throughput biodosimetry test in treatment prioritization after a nuclear incident. *Int. J. Radiat. Biol*. 1–9, 10.1080/09553002.2018.1532615 (2018). 10.1080/09553002.2018.1532615 30507310

[4] Rodrigues MA, Beaton-Green LA, Wilkins RC, Fenech MF (2018). The potential for complete automated scoring of the cytokinesis block micronucleus cytome assay using imaging flow cytometry. Mutat. Res. Toxicol. Environ. Mutagen..

[5] Vral A, Fenech M, Thierens H (2011). The micronucleus assay as a biological dosimeter of *in vivo* ionising radiation exposure. Mutagenesis.

[6] Blakely WF (2016). U.S. Department of Defense Multiple-Parameter Biodosimetry Network. Radiat. Prot. Dosimetry.

[7] Wojcik A (2017). The RENEB operational basis: complement of established biodosimetric assays. Int. J. Radiat. Biol..

[8] Homer MJ (2016). United states department of health and human services biodosimetry and radiological/nuclear medical countermeasure programs PROGRAMS. Radiat. Prot. Dosimetry.

[9] Coleman CN, Koerner JF (2016). Biodosimetry: Medicine, Science, and Systems to Support the Medical Decision-Maker Following a Large Scale Nuclear or Radiation Incident. Radiat. Prot. Dosimetry.

[10] Milner EE (2016). Concepts of Operations (CONOPS) for Biodosimetry Tools Employed in Operational Environments. Health Phys..

[11] Garty G, Karam A, Brenner DJ (2011). Infrastructure to support ultra high throughput biodosimetry screening after a radiological event. Int. J. Radiat. Biol..

[12] Grace MB (2010). Rapid radiation dose assessment for radiological public health emergencies: roles of NIAID and BARDA. Health Phys..

[13] Akiba S (2012). Epidemiological studies of Fukushima residents exposed to ionising radiation from the Fukushima Daiichi Nuclear Power Plant prefecture–a preliminary review of current plans. J. Radiol. Prot..

[14] Cullings HM (2006). Dose estimation for atomic bomb survivor studies: its evolution and present status. Radiat. Res..

[15] Defense_Threat_Reduction_Agency. *Monte Carlo Modeling of the Initial Radiation Emitted by an Improvised Nuclear Device in the National Capital Region (Revision 1). Report No. DTRA-TR-13*-045. (2016).

[16] Mitchell CR (2004). Stable intrachromosomal biomarkers of past exposure to densely ionizing radiation in several chromosomes of exposed individuals. Radiat. Res..

[17] Brenner DJ (2001). Biomarkers specific to densely-ionising (high LET) radiations. Radiat. Prot. Dosimetry.

[18] Ryan TL (2019). Optimization and validation of automated dicentric chromosome analysis for radiological/nuclear triage applications. Mutat. Res. Toxicol. Environ. Mutagen..

[19] Kang CM, Yun HJ, Kim H, Kim CS (2016). Strong correlation among three biodosimetry techniques following exposures to ionizing radiation. Genome Integr..

[20] Lue SW, Repin M, Mahnke R, Brenner DJ (2015). Development of a High-Throughput and Miniaturized Cytokinesis-Block Micronucleus Assay for Use as a Biological Dosimetry Population Triage Tool. Radiat. Res..

[21] Terzoudi GI (2017). Dose assessment intercomparisons within the RENEB network using G _0_ -lymphocyte prematurely condensed chromosomes (PCC assay). Int. J. Radiat. Biol..

[22] Garty G (2015). An automated imaging system for radiation biodosimetry. Microsc. Res. Tech..

[23] Homeland_Security_Council. *National Planning Scenarios (Final Version 21*.3). (2006).

[24] Egbert SD, Kerr GD, Cullings HM (2007). DS02 fluence spectra for neutrons and gamma rays at Hiroshima and Nagasaki with fluence-to-kerma coefficients and transmission factors for sample measurements. Radiat. Environ. Biophys..

[25] Stricklin, D., Kramer, K. & Prins, R. *Review of Deterministic Neutron RBEs for Survivable Personnel Radiation Exposures from Nuclear Detonation Simulations*. (2018).

[26] Xu Y (2015). Accelerator-Based Biological Irradiation Facility Simulating Neutron Exposure from an Improvised Nuclear Device. Radiat. Res..

[27] Wuttke K, Müller WU, Streffer C (1998). The sensitivity of the *in vitro* cytokinesis-blocked micronucleus assay in lymphocytes for different and combined radiation qualities. Strahlenther. Onkol..

[28] Laiakis EC (2019). Serum lipidomic analysis from mixed neutron/X-ray radiation fields reveals a hyperlipidemic and pro-inflammatory phenotype. Sci. Rep..

[29] Broustas CG, Harken AD, Garty G, Amundson SA (2018). Identification of differentially expressed genes and pathways in mice exposed to mixed field neutron/photon radiation. BMC Genomics.

[30] Cary LH, Ngudiankama BF, Salber RE, Ledney GD, Whitnall MH (2012). Efficacy of radiation countermeasures depends on radiation quality. Radiat. Res..

[31] Glasstone, S. & Dolan, P. *The Effects of Nuclear Weapons. Third edition*. (1977).

[32] Eslinger PW, Bowyer TW, Cameron IM, Hayes JC, Miley HS (2015). Atmospheric plume progression as a function of time and distance from the release point for radioactive isotopes. J. Environ. Radioact..

[33] Jackson IL (2019). Hematological Effects of Non-Homogenous Ionizing Radiation Exposure in a Non-Human Primate Model. Radiat. Res..

[34] Geraci JP, Jackson KL, Mariano MS, Michieli BM (1990). Kidney and Lung Injury in Irradiated Rats Protected from Acute Death by Partial-Body Shielding. Radiat. Res..

[35] van Bekkum DW, Schotman E (1974). Protection from haemopoietic death by shielding versus grafting of bone-marrow. Int. J. Radiat. Biol. Relat. Stud. Phys. Chem. Med..

[36] Shea-Donohue T (2016). Mechanisms Involved in the Development of the Chronic Gastrointestinal Syndrome in Nonhuman Primates after Total-Body Irradiation with Bone Marrow Shielding. Radiat. Res..

[37] Oliveira M (2016). Zero-inflated regression models for radiation-induced chromosome aberration data: A comparative study. Biometrical J..

[38] Ainsbury EA (2017). Uncertainty of fast biological radiation dose assessment for emergency response scenarios. Int. J. Radiat. Biol..

[39] Sproull MT, Camphausen KA, Koblentz GD (2017). Biodosimetry: A Future Tool for Medical Management of Radiological Emergencies. Heal. Secur..

[40] Higueras M, Puig P, Ainsbury EA, Rothkamm K (2015). A new inverse regression model applied to radiation biodosimetry. Proceedings. Math. Phys. Eng. Sci..

[41] Moriña D, Higueras M, Puig P, Ainsbury EA, Rothkamm K (2015). radir package: an R implementation for cytogenetic biodosimetry dose estimation. J. Radiol. Prot..

[42] Ainsbury EA (2014). Review of Bayesian statistical analysis methods for cytogenetic radiation biodosimetry, with a practical example. Radiat. Prot. Dosimetry.

[43] Higueras, M. & Ainsbury, E. A. Bayesian Solutions to Biodosimetry Count Data Problems and Supporting Software. in 103–107, 10.1007/978-3-319-55639-0_17 (2017).

[44] Sproull M, Camphausen K (2016). State-of-the-Art Advances in Radiation Biodosimetry for Mass Casualty Events Involving Radiation Exposure. Radiat. Res..

[45] Mendes, M. E. *et al*. Comparative study of Micronucleus assays and Dicentric plus ring chromosomes for dose assessment in particular cases of partial-body exposure. *Int. J. Radiat. Biol*. 1–36, 10.1080/09553002.2019.1607606 (2019). 10.1080/09553002.2019.1607606 31008687

[46] Słonecka I, Łukasik K, Fornalski KW (2019). Simplified Bayesian method: application in cytogenetic biological dosimetry of mixed n + γ radiation fields. Radiat. Environ. Biophys..

[47] Słonecka I, Łukasik K, Fornalski KW (2018). Analytical and quasi-Bayesian methods as development of the iterative approach for mixed radiation biodosimetry. Radiat. Environ. Biophys..

[48] Grégoire E (2018). Twenty years of FISH-based translocation analysis for retrospective ionizing radiation biodosimetry. Int. J. Radiat. Biol..

[49] Liu J (2017). Accurate cytogenetic biodosimetry through automated dicentric chromosome curation and metaphase cell selection. F1000Research.

[50] Oestreicher U (2017). RENEB intercomparisons applying the conventional Dicentric Chromosome Assay (DCA). Int. J. Radiat. Biol..

[51] Barquinero JF (2017). RENEB biodosimetry intercomparison analyzing translocations by FISH. Int. J. Radiat. Biol..

[52] Depuydt J (2017). RENEB intercomparison exercises analyzing micronuclei (Cytokinesis-block Micronucleus Assay). Int. J. Radiat. Biol..

[53] Beaton-Green LA, Barr T, Ainsbury EA, Wilkins RC (2016). Retrospective Biodosimetry of an Occupational Overexposure—Case Study. Radiat. Prot. Dosimetry.

[54] Williams BB, Flood AB, Demidenko E, Swartz HM (2016). ROC Analysis for Evaluation of Radiation Biodosimetry Technologies. Radiat. Prot. Dosimetry.

[55] Rodrigues MA, Probst CE, Beaton-Green LA, Wilkins RC (2016). Optimized automated data analysis for the cytokinesis-block micronucleus assay using imaging flow cytometry for high throughput radiation biodosimetry. Cytom. Part A.

[56] Beinke C, Port M, Riecke A, Ruf CG, Abend M (2016). Adaption of the Cytokinesis-Block Micronucleus Cytome Assay for Improved Triage Biodosimetry. Radiat. Res..

[57] Balog, R. P. *et al*. Development of a point-of-care radiation biodosimeter: studies using novel protein biomarker panels in non-human primates. *Int. J. Radiat. Biol*. 1–12, 10.1080/09553002.2018.1532612 (2018). 10.1080/09553002.2018.1532612 30394814

[58] Turner HC (2011). Adapting the gamma-H2AX assay for automated processing in human lymphocytes. 1. Technological aspects. Radiat. Res..

[59] Pannkuk EL (2016). A Lipidomic and Metabolomic Serum Signature from Nonhuman Primates Exposed to Ionizing Radiation. Metabolomics.

[60] Laiakis EC (2014). Development of a Metabolomic Radiation Signature in Urine from Patients Undergoing Total Body Irradiation. Radiat. Res..

[61] Park JG (2017). Developing Human Radiation Biodosimetry Models: Testing Cross-Species Conversion Approaches Using an *Ex Vivo* Model System. Radiat. Res..

[62] Ossetrova, N. I. *et al*. Biomarkers for Radiation Biodosimetry and Injury Assessment after Mixed-field (Neutron and Gamma) Radiation in the Mouse Total-body Irradiation Model. *Health Phys*. 1, 10.1097/HP.0000000000000938 (2018). 10.1097/HP.0000000000000938 30299338

[63] Macaeva E, Mysara M, De Vos WH, Baatout S, Quintens R (2019). Gene expression-based biodosimetry for radiological incidents: assessment of dose and time after radiation exposure. Int. J. Radiat. Biol..

[64] Lee Y (2018). Candidate protein markers for radiation biodosimetry in the hematopoietically humanized mouse model. Sci. Rep..

[65] Entine F (2018). Deployment of the DosiKit System Under Operational Conditions. Health Phys..

[66] Swarts SG (2018). Developments in Biodosimetry Methods for Triage With a Focus on X-band Electron Paramagnetic Resonance *In Vivo* Fingernail Dosimetry. Health Phys..

[67] Kiang JG, Smith JT, Hegge SR, Ossetrova NI (2018). Circulating Cytokine/Chemokine Concentrations Respond to Ionizing Radiation Doses but not Radiation Dose Rates: Granulocyte-Colony Stimulating Factor and Interleukin-18. Radiat. Res..

[68] Tomasik B, Fendler W, Chowdhury D (2018). Serum microRNAs &amp;#x2013; potent biomarkers for radiation biodosimetry. Oncotarget.

[69] Zeegers D (2017). Biomarkers of ionizing radiation exposure: A multiparametric approach. Genome Integr..

[70] Shuryak, I., Loucas, B. D. & Cornforth, M. N. Straightening Beta: Overdispersion of Lethal Chromosome Aberrations following Radiotherapeutic Doses Leads to Terminal Linearity in the Alpha–Beta Model. *Front. Oncol*. **7**, (2017). 10.3389/fonc.2017.00318 PMC5742594 29312888

[71] Ainsbury EA (2013). CytoBayesJ: Software tools for Bayesian analysis of cytogenetic radiation dosimetry data. Mutat. Res. Toxicol. Environ. Mutagen..

[72] Higueras M, González JE, Di Giorgio M, Barquinero JF (2018). A note on Poisson goodness-of-fit tests for ionizing radiation induced chromosomal aberration samples. Int. J. Radiat. Biol..

[73] Edwards AA, Lloyd DC, Purrott RJ (1979). Radiation induced chromosome aberrations and the Poisson distribution. Radiat. Environ. Biophys..

[74] Schmid E, Hieber L, Heinzmann U, Roos H, Kellerer AM (1996). Analysis of chromosome aberrations in human peripheral lymphocytes induced by *in vitro* alpha-particle irradiation. Radiat. Environ. Biophys..

[75] Szłuińska, M., Edwards, A. A. & Lloyd, D. C. Statistical Methods for Biological Dosimetry. in *Chromosomal Alterations* 351–370 (Springer Berlin Heidelberg). 10.1007/978-3-540-71414-9_23.

[76] Barquinero JF (1997). Biological dosimetry in simulated *in vitro* partial irradiations. Int. J. Radiat. Biol..

[77] Senthamizhchelvan S, Pant GS, Rath GK, Julka PK, Nair O (2009). Biodosimetry using micronucleus assay in acute partial body therapeutic irradiation. Phys. Medica.

[78] Dolphin, G. *Biological dosimetry with particular reference to chromosome aberration analysis. A review of methods*. (1969).

[79] Romero I (2013). Assessment of simulated high-dose partial-body irradiation by PCC-R assay. J. Radiat. Res..

[80] Horn S, Barnard S, Rothkamm K (2011). Gamma-H2AX-Based Dose Estimation for Whole and Partial Body Radiation Exposure. Plos One.

[81] Pittman, B. *et al*. Models for Analyzing Zero-Inflated and Overdispersed Count Data: An Application to Cigarette and Marijuana Use. *Nicotine Tob. Res*., 10.1093/ntr/nty072 (2018). 10.1093/ntr/nty072 PMC7364829 29912423

[82] Zhou M, Carin L (2015). Negative Binomial Process Count and Mixture Modeling. IEEE Trans. Pattern Anal. Mach. Intell..

[83] Zhao JZL, Mucaki EJ, Rogan PK (2018). Predicting ionizing radiation exposure using biochemically-inspired genomic machine learning. F1000Research.

[84] LI Y, Knoll JH, Wilkins RC, Flegal FN, Rogan PK (2016). Automated discrimination of dicentric and monocentric chromosomes by machine learning-based image processing. Microsc. Res. Tech..

[85] Holzinger, E. R. *et al*. Variable selection method for the identification of epistatic models. pmcid: PMC4299919. *Pac. Symp. Biocomput*. 195–206 (2015). PMC4299919 25592581

[86] Hastie, T., Tibshirani, R. & Friedman, J. H. *The elements of statistical learning: data mining, inference, and prediction*. 10.1007/BF02985802. (Springer, 2009).

[87] Bian D (2016). Liquid Handling Optimization in High-Throughput Biodosimetry Tool. J. Med. Device..

[88] Xu Y (2012). Novel neutron sources at the Radiological Research Accelerator Facility. J. Instrum..

[89] Xu Y (2015). Broad Energy Range Neutron Spectroscopy using a Liquid Scintillator and a Proportional Counter: Application to a Neutron Spectrum Similar to that from an Improvised Nuclear Device. Nucl. Instrum. Methods Phys. Res. A..

[90] Swanson RK, Xu R, Nettleton D, Glatz CE (2012). Proteomics-based, multivariate random forest method for prediction of protein separation behavior during cation-exchange chromatography. J. Chromatogr. A.

[91] Hengl T (2018). Global mapping of potential natural vegetation: an assessment of machine learning algorithms for estimating land potential. PeerJ.

[92] Swets JA, Dawes RM, Monahan J (2000). Psychological Science Can Improve Diagnostic Decisions. Psychol. Sci. Public Interes..

[93] Huber R, Schraube H, Nahrstedt U, Braselmann H, Bauchinger M (1994). Dose-response relationships of micronuclei in human lymphocytes induced by fission neutrons and by low LET radiations. Mutat. Res..

[94] Ryan TL, Pantelias AG, Terzoudi GI, Pantelias GE, Balajee AS (2019). Use of human lymphocyte G0 PCCs to detect intra- and inter-chromosomal aberrations for early radiation biodosimetry and retrospective assessment of radiation-induced effects. PLoS One.

[95] Smith T (2019). Extension of lymphocyte viability for radiation biodosimetry: Potential implications for radiological/nuclear mass casualty incidents. J. Cell. Biochem..

[96] Balajee AS, Hande MP (2018). History and evolution of cytogenetic techniques: Current and future applications in basic and clinical research. Mutat. Res. Toxicol. Environ. Mutagen..

[97] Repin M, Pampou S, Karan C, Brenner DJ, Garty G (2017). RABiT-II: Implementation of a High-Throughput Micronucleus Biodosimetry Assay on Commercial Biotech Robotic Systems. Radiat. Res..

[98] Royba E (2019). RABiT-II-DCA: A Fully-automated Dicentric Chromosome Assay in Multiwell Plates. Radiat. Res..

[99] Székely GJ, Rizzo ML (2004). Mean distance test of Poisson distribution. Stat. Probab. Lett..

